# Frondoside A Enhances the Anti-Cancer Effects of Oxaliplatin and 5-Fluorouracil on Colon Cancer Cells

**DOI:** 10.3390/nu10050560

**Published:** 2018-05-01

**Authors:** Samir Attoub, Kholoud Arafat, Tamam Khalaf, Shahrazad Sulaiman, Rabah Iratni

**Affiliations:** 1 Department of Pharmacology & Therapeutics, College of Medicine & Health Sciences, United Arab Emirates University, Al-Ain P.O. Box 17666, UAE; kholoud.arafat@uaeu.ac.ae (K.A.); tamam.es.khalaf@gmail.com (T.K.); sharazadjeffy@uaeu.ac.ae (S.S.); 2 Institut National de la Santé et de la Recherche Médicale (INSERM), 75571 Paris Cedex 12, France; 3 Department of Biology, College of Science, United Arab Emirates University, Al-Ain P.O. Box 15551, UAE; R_iratni@uaeu.ac.ae

**Keywords:** colon cancer, Frondoside A, oxaliplatin, 5-fluorouracil, cell proliferation, apoptosis

## Abstract

Over recent years, we have demonstrated that Frondoside A, a triterpenoid glycoside isolated from an Atlantic sea cucumber, has potent in vitro and in vivo anti-cancer effects against human pancreatic, breast, and lung cancer. We have also demonstrated that Frondoside A is able to potentiate and/or synergize the anti-cancer effects of major classical cytotoxic agents, namely, gemcitabine, paclitaxel, and cisplatin, in the treatment of pancreatic, breast, and lung cancer, respectively. This study evaluates the impact of Frondoside A alone and in combination with the standard cytotoxic drugs oxaliplatin and 5-fluorouracil (5-FU) in the treatment of colon cancer using three human colon cancer cell lines, namely, HT-29, HCT-116, and HCT8/S11. We demonstrate that Frondoside A, oxaliplatin, and 5-FU cause a concentration- and time-dependent reduction in the number of HT-29 colon cancer cells. A concentration of 2.5 µM of Frondoside A led to almost 100% inhibition of cell numbers at 72 h. A similar effect was only observed with a much higher concentration (100 µM) of oxaliplatin or 5-FU. The reduction in cell numbers by Frondoside A, oxaliplatin, and 5-FU was also confirmed in two other colon cancer cell lines, namely, HCT8/S11 and HCT-116, treated for 48 h. The combinations of low concentrations of these drugs for 48 h in vitro clearly demonstrated that Frondoside A enhances the inhibition of cell numbers induced by oxaliplatin or 5-FU. Similarly, such a combination also efficiently inhibited colony growth in vitro. Interestingly, we found that the inhibition of ERK1/2 phosphorylation was significantly enhanced when Frondoside A was used in combination treatments. Moreover, we show that Frondoside A and 5-FU, when used alone, induce a concentration-dependent induction of apoptosis and that their pro-apoptotic effect is dramatically enhanced when used in combination. We further demonstrate that apoptosis induction upon the treatment of colon cancer cells was at least in part a result of the inhibition of phosphorylation of the survival kinase AKT, leading to caspase-3 activation, poly (ADP-ribose) polymerase (PARP) inactivation, and consequently DNA damage, as suggested by the increase in the level of γH2AX. In light of these findings, we strongly suggest that Frondoside A may have a role in colon cancer therapy when used in combination with the standard cytotoxic drugs oxaliplatin and 5-FU.

## 1. Introduction

Colorectal cancer is the third leading cause of cancer death in the world and represents a serious threat to human health [1]. Without treatment, patients with inoperable or metastatic colorectal cancer have a median life expectancy of about 8 months. The standard cytotoxic drugs for the treatment of metastatic colorectal cancer are 5-fluorouracil (5-FU) (combined with folinic acid), oxaliplatin, and irinotecan. These chemotherapeutic agents currently in use for colon cancer remain unsatisfactory because of their associated collateral toxicity and resistance. In the last decade, the survival rate of patients with metastatic colorectal cancer has improved with the application of targeted drugs such as Bevacizumab. Despite these advances, patients still die, and a cure remains elusive [2].

Patients, oncologists, and scientists have for decades expressed interest in using natural compound remedies because of the overall disappointing results and side effects of the current cytotoxic drugs and targeted therapies. Recent studies have demonstrated that low concentrations of Frondoside A, a triterpenoid glycoside isolated from the Atlantic cucumber *Cucumaria frondosa*, induces apoptosis in human pancreatic, leukemic, breast, lung, and prostate cancer cells, leading to the inhibition of their tumor xenograft growth in vivo [3,4,5,6,7]. Investigators have also demonstrated that Frondoside A was able to synergize or to potentiate the anti-cancer effects of major classical cytotoxic agents, namely, gemcitabine, paclitaxel, and cisplatin, in the treatment of pancreatic [8], breast [5], and lung [6] cancer xenografts, respectively.

We know from clinical trials that single-agent treatments rarely result in clinical benefits to cancer patients and that combination therapy is necessary for the effective treatment of tumors. This study investigates the potential anti-cancer effects of Frondoside A alone and in combination with the standard cytotoxic drugs oxaliplatin and 5-FU (FOLFOX protocol) in the treatment of colon cancer.

## 2. Materials and Methods

### 2.1. Cell Culture and Reagents

The human colon cancer cell lines HT-29, HCT-116, and HCT8/S11 were maintained in DMEM (Hyclone, Cramlington, UK) supplemented with antibiotics (penicillin: 50 U/mL; streptomycin: 50 µg/mL) (Hyclone, Cramlington, UK) and with 10% fetal bovine serum (FBS; Biowest, Nouaille, France). In all experiments, the cell viability was higher than 99% using trypan blue dye exclusion. Frondoside A, oxaliplatin, and 5-FU were purchased from Sigma-Aldrich (Sigma-Aldrich, Saint Louis, MO, USA). Antibodies to phospho-AKT, phospho-p44/42 MAPK (ERK1/2), cleaved caspase-3 (Asp175), and cleaved poly (ADP-ribose) polymerase (PARP) were obtained from Cell Signaling Technology (Cell Signaling, Beverly, MA, USA). The AKT antibody was obtained from Abcam (Abcam, Cambridge, UK). The antibody to phospho-histone H2AX was obtained from Millipore (Millipore, Hayward, CA, USA). Antibodies to ERK2 and β-actin were obtained from Santa Cruz Biotechnology, Inc. (Santa Cruz, CA, USA).

### 2.2. Impact of Frondoside A, Oxaliplatin, 5-Fluorouracil, and Their Combinations on Colon Cancer Cell Numbers

Cells were seeded at a density of 50,000 cells per well into 6-well plates. After 24 h, the cells were treated for another 24, 48, and 72 h with increasing concentrations of Frondoside A (0.1–5 µM), oxaliplatin (1–100 µM), or 5-FU (1–100 µM) in triplicate. Control cultures were treated with 0.1% DMSO (the drug vehicle). In the second set of experiments, cells were treated with a combination of Frondoside A and oxaliplatin or Frondoside A and 5-FU. The effects of these combinations on the cell numbers were determined at the indicated times using the Scepter 2.0 Handheld Automated Cell Counter (Millipore, Hayward, CA, USA). Data were presented as the proportional cell numbers (%) by comparing the drug-treated cells with the DMSO-treated cells, the cell numbers of which were assumed to be 100%.

### 2.3. Impact of the Treatments on Colony Growth in Matrigel Matrix

A layer of 150 µL of matrigel was poured into the wells of a 24-well cell culture dish and allowed to set at 37 °C for 30 min. A second layer (300 µL) composed of 150 µL of matrigel dissolved in 150 µL of growth media containing 1.5 × 10^3^ cells was placed on top of the first layer and allowed to set in the humidified incubator at 37 °C for 30 min. Growth medium (0.5 mL) was added on top of the second layer, and the cells were incubated in a humidified incubator at 37 °C for 14 days and then treated for another 7 days with Frondoside A, oxaliplatin, 5-FU, or the combinations. Control cells were exposed to 0.1% DMSO. The medium was changed twice a week. At the end of the experiment, colonies were stained for 1 h with 2% Giemsa stain and incubated with PBS overnight to remove excess Giemsa stain. The colonies were photographed and scored, and the percentages of colonies larger than 100 µm were determined. Data presented compare the drug-treated colonies with the DMSO-treated colonies. Colonies larger than 100 µm were expressed as a percentage of the total counted colonies and were then compared to the DMSO-treated controls.

### 2.4. Quantification of Apoptosis by Annexin V Labeling

Apoptosis was examined using the Annexin V Dead Cell kit (Millipore, Hayward, CA, USA) according to the manufacturer’s instructions. Briefly, HT-29 cells were treated with or without each compound individually or in combination for 48 h. Detached and adherent cells were collected and incubated with Annexin V and 7-AAD, a dead cell marker, for 20 min at room temperature in the dark. The early and late apoptotic cells were counted with the Muse Cell Analyzer (Millipore, Hayward, CA, USA). Experiments were carried out in triplicate and were repeated three times.

### 2.5. Impact of Frondoside A, Oxaliplatin, 5-Fluorouracil, and Their Combinations on Pro-Apoptotic and Anti-Proliferation Proteins’ Expression and Phosphorylation Levels

Cells were seeded in 100 mm dishes at 2 × 10^6^ cells per dish for 24 h and were then treated with Frondoside A (0.5 µM), oxaliplatin (10 µM), 5-FU (10 µM), a combination of Frondoside A and oxaliplatin, or Frondoside A and 5-FU, for another 24 h. Control cultures were treated with 0.1% DMSO (the drug vehicle). In a second set of experiments, cells were treated with increasing concentrations of Frondoside A (0.5–2.5 µM) for 2 h. Total cellular proteins were isolated using a RIPA buffer (25 mM Tris.HCl, pH 7.6; 1% Nonidet P-40; 1% sodium deoxycholate; 0.1% SDS; 0.5% protease inhibitor cocktail; 1% PMSF; 1% phosphatase inhibitor cocktail) from the DMSO- and drug-treated cells. The whole cell lysates were recovered by centrifugation at 14,000 rpm for 20 min at 4 °C to remove insoluble material, and the protein concentrations of the lysates were determined using a BCA protein assay kit (Thermo Fisher Scientific, Waltham, MA, USA). Proteins (30 µg) were separated by SDS-PAGE gel to determine the expression and the phosphorylation levels of different pro-apoptotic and anti-proliferation proteins (AKT, p-AKT, activated caspase-3, cleaved PARP, p-H2AX, ERK-2, p-ERK, and β-actin). After electrophoresis, the proteins were transferred onto a nitrocellulose membrane, blocked for 1 h at room temperature with 5% non-fat milk in TBST (TBS and 0.05% Tween 20), and then probed with specific primary antibodies and β-actin (1:1000) overnight at 4 °C. The blots were washed, exposed to secondary antibodies, and visualized using the ECL system (Thermo Fisher Scientific, Waltham, MA, USA). Membrane stripping was performed by incubating the membrane in Restore Western blot stripping buffer (Thermo Fisher Scientific, Waltham, MA, USA) according to the manufacturer’s instructions. Densitometry analysis was performed using an HP Deskjet F4180 Scanner (HP Development Company, Palo Alto, CA, USA) with ImageJ software.

### 2.6. Statistics

Results are expressed as means ± S.E.M. of the indicated data. The difference between the experimental and control values was assessed by ANOVA followed by Dunnett’s post hoc multiple comparison test (*** *p* < 0.001, ** *p* < 0.01, and * *p* < 0.05 indicate a significant difference).

## 3. Results and Discussion

### 3.1. Effect of Frondoside A, Oxaliplatin and 5-Fluorouracil on Cell Numbers

**Figure 1:**
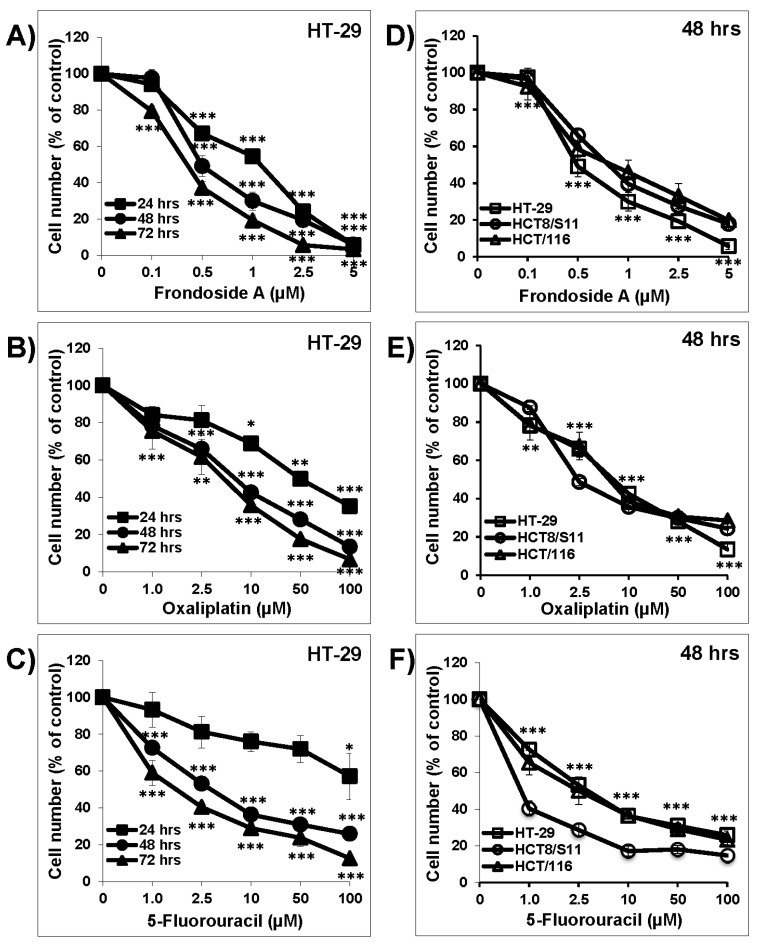
Frondoside A inhibition of colon cancer cell numbers compared to oxaliplatin and 5-fluorouracil. Exponentially growing HT-29 cells were treated with vehicle (0.1% DMSO) and the indicated concentrations of Frondoside A (**A**), oxaliplatin (**B**), and 5-fluorouracil (**C**) for 24 to 72 h. The effects of the three drugs were confirmed at 48 h in two additional colon cancer cell lines, namely, HCT8/S11 and HCT-116 (**D**–**F**). Cell count was determined as described in Materials and Methods. All experiments were repeated at least three times. Shapes represent means; bars represent S.E.M. * Significantly different at *p* < 0.05. ** Significantly different at *p* < 0.01. *** Significantly different at *p* < 0.001.

As shown in Figure 1, Frondoside A (0.1–5 µM), oxaliplatin (1–100 µM), and 5-FU (1–100 µM) caused a concentration- and time-dependent decrease in HT-29 cell numbers over 24, 48, and 72 h (Figure 1A–C). The concentration-dependent effects of Frondoside A, oxaliplatin, and 5-FU were also confirmed on two other colon cancer cell lines, namely, HCT-116 and HCT8/S11 (Figure 1D–F). At 48 h, the IC_50_ concentration of Frondoside A was 0.5 µM in HT-29 cells and approximately 0.75 µM in both HCT-116 and HCT8/S11 cells (Table 1). On the other hand, the IC_50_ concentrations of oxaliplatin and 5-FU were found to be much higher than for Frondoside A (Table 1). Because previous work has reported the anti-tumor activity of oxaliplatin and 5-FU in HT-29 colon cancer cells [9], we therefore decided to pursue the rest of this study on HT-29 cells.

### 3.2. Frondoside A Enhanced the Anti-Tumor Activity of Oxaliplatin and 5-Fluorouracil on HT-29 in Cell Count and Colony Growth Assays

**Figure 2:**
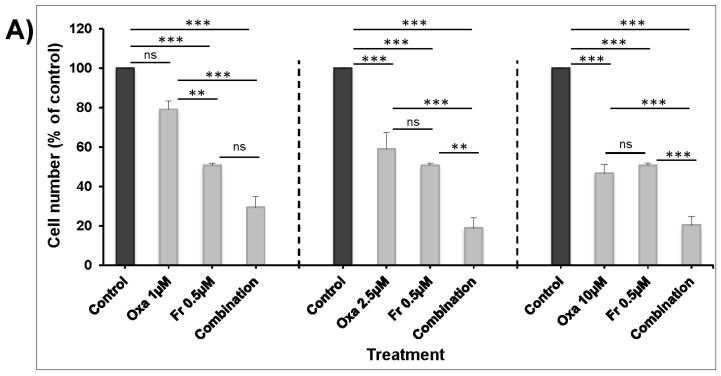

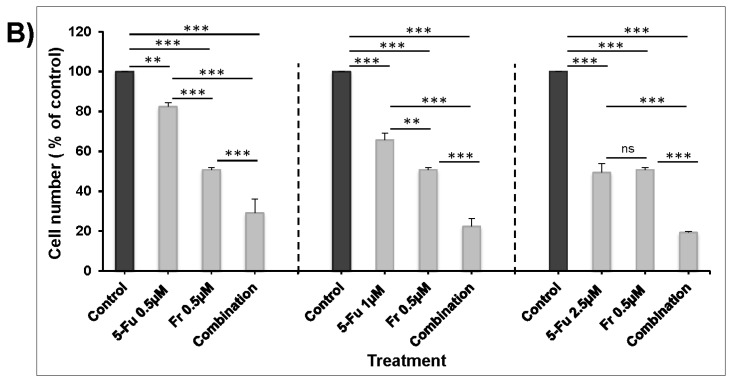
Frondoside A enhances the inhibition of HT-29 cell numbers by (**A**) oxaliplatin (1–10 μM), and (**B**) 5-fluorouracil (0.5–2.5 μM). Cells were treated for 48 h, and all experiments were repeated at least three times. Columns are means; bars are S.E.M. ** Significantly different at *p* < 0.01. *** Significantly different at *p* < 0.001. ns (not significant).

**Figure 3:**
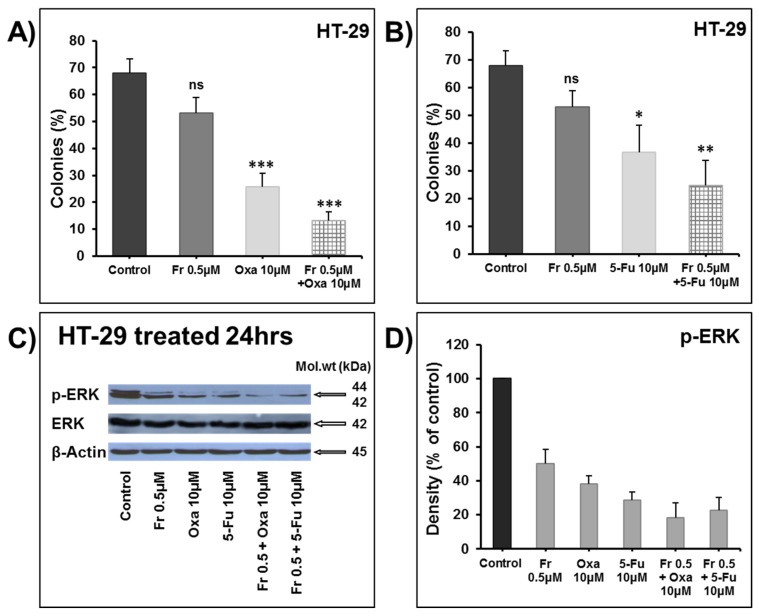
Frondoside A enhances the inhibition of colonies’ growth by (**A**) oxaliplatin (10 µM), and (**B**) 5-fluorouracil (10 µM). Data are presented as histograms of the mean percentage of large colonies’ growth ± S.E.M. (**C**) The inhibition of ERK phosphorylation by oxaliplatin and 5-fluorouracil was enhanced by Frondoside A in HT-29 colon cancer cells. (**D**) Densitometry analysis of p-ERK from three different experiments. β-actin was used as an internal loading control of the protein levels, and the normalized p-ERK bands’ densities are expressed as percentage change in comparison to control samples considered equal to 100%. * Significantly different at *p* < 0.05. ** Significantly different at *p* < 0.01. *** Significantly different at *p* < 0.001. ns (not significant).

The treatment of HT-29 cells for 48 h using the IC_50_ concentration of Frondoside A (0.5 μM) significantly enhanced the growth inhibitory effects of increasing concentrations of oxaliplatin (1–10 μM) (Figure 2A) and 5-FU (0.5–2.5 μM) (Figure 2B). We next examined the impact of these combinations on the growth of already formed HT-29 colonies. First, HT-29 cells were allowed to grow and form visible colonies in the absence of any treatment. After two weeks of growth, the colonies were treated for one more week with DMSO as the control, Frondoside A (0.5 μM), oxaliplatin (10 μM), 5-FU (10 μM), or a combination. Although the number of colonies obtained in each treatment seemed unchanged, we noticed, however, that the sizes of the colonies were significantly reduced. Indeed, while large colonies represented approximately 70% of the total number of colonies in the control, they represented only 25% and 35% of the total number of colonies exposed to both oxaliplatin (Figure 3A) and 5-FU (Figure 3B) alone. Although Frondoside A did not lead to significant colony growth inhibition when compared to oxaliplatin or 5-FU alone, when used in combination, Frondoside A significantly enhanced the inhibition of colony growth mediated by oxaliplatin (Figure 3A) or 5-FU (Figure 3B). We speculate that the effect of these drugs alone and in combination on the size of the colony growth may be due to cell death and/or inhibition of cellular proliferation.

It is well documented that the MAPK signaling pathway is mainly involved in regulating cellular proliferation and that a blockade of this pathway suppresses the growth of colon tumors [10]. ERK1 and ERK2, the final effectors of the MAPK pathway, activated through phosphorylation, lead to the activation of a variety of substrates responsible for the induction of cell proliferation. Hence, we decided to investigate the activation of ERK1/2 in response to Frondoside A alone and in combination with oxaliplatin and 5-FU. Interestingly, although we observed a marked inhibition of ERK1/2 phosphorylation with single treatments, a combination of the drugs enhanced this inhibition (Figure 3C,D).

### 3.3. Combination of Frondoside A with 5-Fluorouracil or Oxaliplatin Enhances Apoptotic Cell Death in HT-29 Colon Cancer Cells

**Figure 4:**
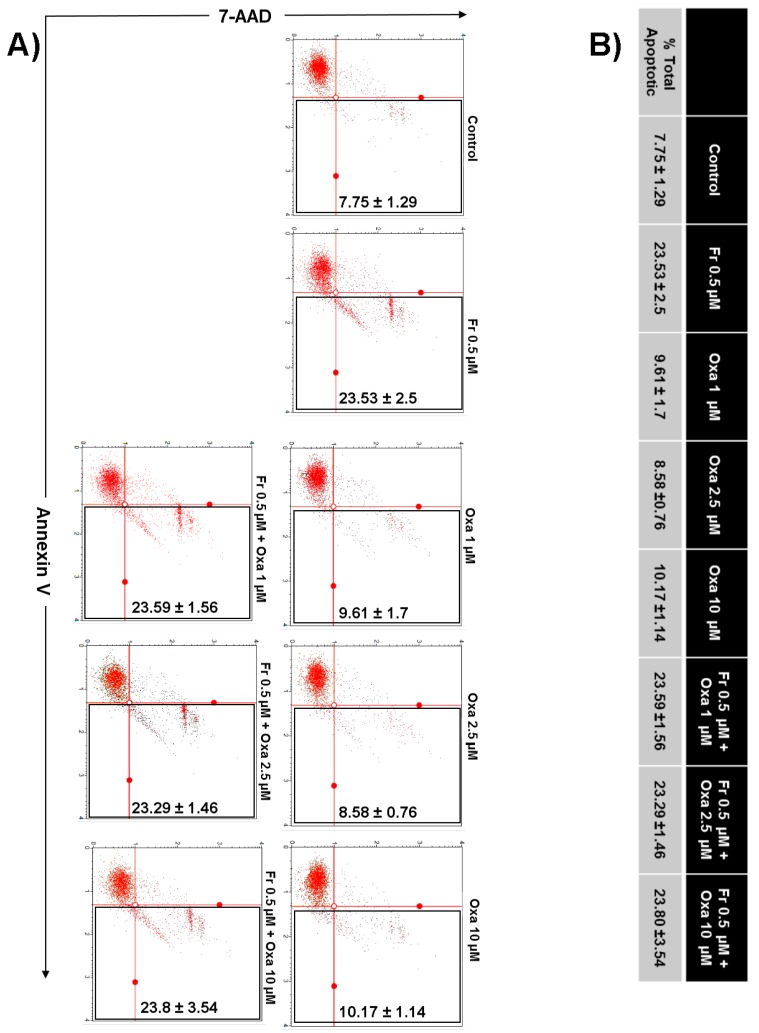
Impact of Frondoside A on apoptotic cell death induced by oxaliplatin in HT-29 colon cancer cells. (**A**,**B**) Annexin V binding was carried out using Annexin V Dead Cell kit. HT-29 cells were treated with or without Frondoside A (0.5 μM) and indicated concentrations of oxaliplatin (1, 2.5, and 10 μM) individually and in combination for 48 h. Detached and adherent cells were collected and stained with Annexin V and 7-AAD, and then the events for early and late apoptotic cells were counted with the Muse Cell Analyzer as described in Materials and Methods. Data represent the mean ± S.E.M. of at least three independent experiments. Fr represents Frondoside A and Oxa represents oxaliplatin.

**Figure 5:**
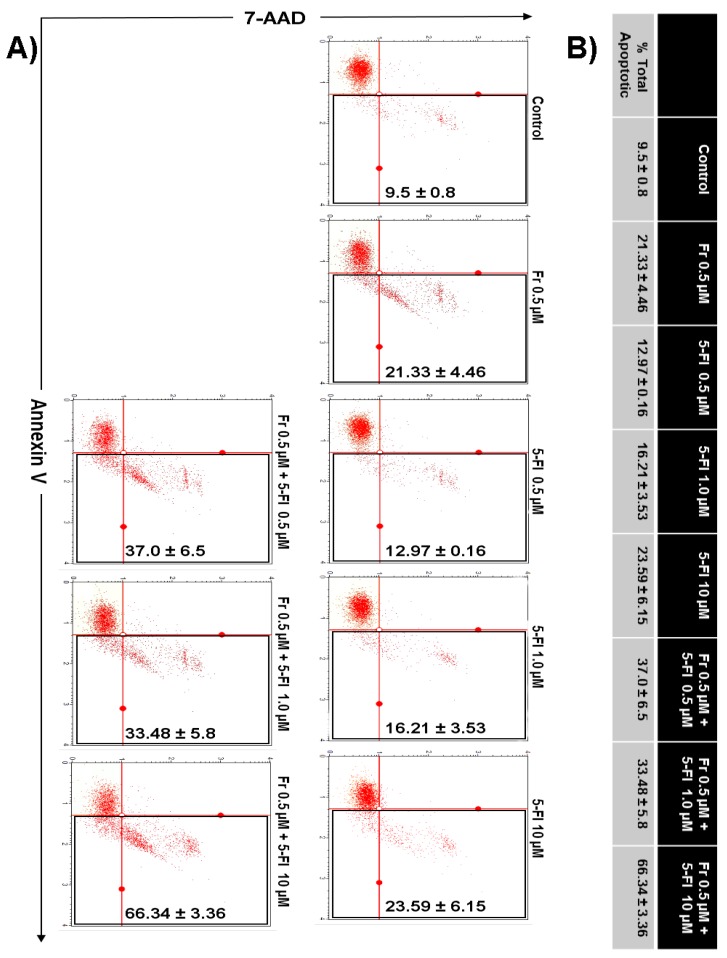
Frondoside A enhances apoptotic cell death induced by 5-fluorouracil in HT-29 colon cancer cells. (**A**,**B**) Annexin V binding was carried out using Annexin V Dead Cell kit. HT-29 cells were treated with or without Frondoside A (0.5 μM) and indicated concentrations of 5-fluorouracil (0.5, 1, and 10 μM) individually and in combination for 48 h. Detached and adherent cells were collected and stained with Annexin V and 7-AAD, and then the events for early and late apoptotic cells were counted with the Muse Cell Analyzer as described in Materials and Methods. Data represent the mean ± S.E.M. of at least three independent experiments. Fr represents Frondoside A and 5-FU represents 5-fluorouracil.

Next, we investigated whether the enhanced decrease in HT-29 cell numbers when Frondoside A was used in combination with 5-FU or oxaliplatin was the result of increased apoptotic cell death. Toward this aim, HT-29 colon cancer cells were incubated with Frondoside A (0.5 µM) alone or in combination with increasing concentrations of oxaliplatin (1–10 μM) or 5-FU (0.5–10 μM), and apoptosis was examined after 48 h using Annexin V staining. As shown in Figure 4, a combination of Frondoside A and oxaliplatin caused no increase in the level of apoptosis in comparison with either drug alone. On the other hand, and very interestingly, we found that a combination of Frondoside A and 5-FU led to an increased number of apoptotic cells. Indeed, the population of apoptotic cells rose from 21% for Frondoside A (0.5 µM) and 23% for 5-FU (10 µM) when used alone to 66% when used in combination, suggesting a possible synergism between Frondoside A and 5-FU at these concentrations (Figure 5).

### 3.4. Effects of Frondoside A, Oxaliplatin, and 5-Fluorouracil alone or in Combination on Survival and Apoptotic Pathways and on the DNA Damage

**Figure 6:**
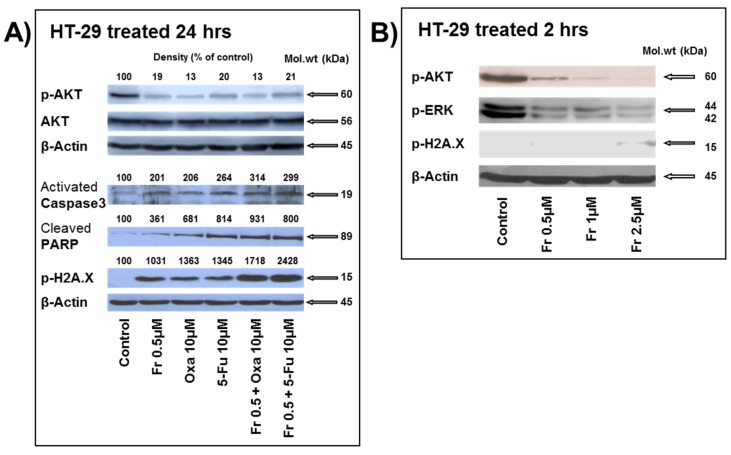
Western blot analysis of: (**A**) the effects of Frondoside A, oxaliplatin, 5-fluorouracil, and their combinations on the phosphorylation of the survival kinase AKT and on caspase-3 activation, Poly (ADP-ribose) polymerase (PARP) inactivation and H2AX phosphorylation. HT-29 cells were treated for 24 h with the indicated concentrations of Frondoside A, oxaliplatin, 5-fluorouracil, and their combinations. Densitometry analysis is from three different experiments. β-actin was used as an internal loading control of the protein levels, and the normalized bands’ densities are expressed as percentage change in comparison to control samples considered equal to 100%. (**B**) The levels of p-AKT, p-ERK, and p-H2AX after 2 h of treatment with Frondoside A (0.5–2.5 µM).

A marked inhibition of AKT phosphorylation was noted with all three drugs when used alone; however, no further decrease in the level of phosphorylation was observed when the drugs were used in combination (Figure 6A). As expected, neither of the treatments carried out affected the level of total AKT proteins (Figure 6A). It has been reported that downregulation of AKT phosphorylation induces caspase-3-dependent apoptosis [11,12]. In line with these reports, oxaliplatin was shown to induce dephosphorylation of AKT, leading to the accumulation of cleaved caspase-3 [13]. We and others have previously reported that Frondoside A induced caspase-3 cleavage in pancreatic, breast, lung, and prostate cancer cells [3,5,6,7]. In this context, apoptosis induced by Frondoside A alone and in combination with oxaliplatin and 5-FU was further assessed by measuring caspase-3 activation and consequently PARP cleavage and inactivation. Cells treated with Frondoside A (0.5 μM), oxaliplatin (10 μM), and 5-FU (10 μM) for 24 h induced the activation of the executioner caspase-3, a key step to induce apoptosis. This activation was further slightly enhanced when Frondoside A was used in combination with either oxaliplatin or 5-FU (Figure 6A). Caspase-3 activation leads to the cleavage and consequently the inactivation of the downstream PARP, a nuclear protein involved in DNA repair and apoptosis. Similarly, downstream PARP cleavage was also observed in the single and combined treatments of HT-29 cells. This is supported by previous reports demonstrating that Frondoside A, oxaliplatin, and 5-FU induce PARP cleavage [3,7,14].

PARP inhibition using PARP inhibitors is known to increase the levels of DNA-damage-associated phosphorylation of H2AX [15]. Phosphorylated H2AX histone (γH2AX) is a marker of DNA double-strand breaks. 5-FU was previously reported to induce DNA damage in colon cancer cells [16]. To check whether the induction of cell death by Frondoside A, oxaliplatin, and 5-FU is associated with DNA damage, HT-29 cells were exposed to the drugs either alone or in combination, and the total proteins were evaluated for γH2AX expression. We found that cells treated with the three drugs alone underwent DNA damage. Interestingly, when the drugs were used in combination, this further increased the level of DNA damage in the treated cells (Figure 6A). To determine whether Frondoside A mediates its effect through DNA damage, the inhibition of ERK1/2, AKT signaling pathways, or a combination, we performed an experiment to measure the levels of DNA damage, p-AKT, and p-ERK1/2 after 2 h of treatment with Frondoside A (0.5–2.5 µM). Figure 6B clearly demonstrates that the concentration of 0.5 µM of Frondoside A used in this study had no effect on the DNA damage, while it strongly reduced the levels of phosphorylated AKT and ERK. DNA damage occurred at a minimal level only at a very high concentration of Frondoside A. Hence, this result strongly suggests that the inhibition of ERK and AKT signaling is an early event that occurs in response to Frondoside A treatment and that DNA damage is a late event that might result from excessive DNA damage as a consequence of active apoptosis. Taken together, our results strongly suggest that Frondoside A, when used in combination, enhances the anti-proliferative and the pro-apoptotic effects of oxaliplatin and 5-FU.

## 4. Conclusions

To the best of our knowledge, the present study identifies for the first time that Frondoside A treatment significantly inhibits proliferation and induces apoptosis in colon cancer cells. The combinations of Frondoside A with the DNA-damaging agent oxaliplatin or with the thymidylate synthase inhibitor 5-FU were significantly more effective in inhibiting HT-29 cell proliferation and triggering apoptosis, leading to the inhibition of the HT-29 colonies’ growth, than either cytotoxic agent alone.

Our data suggests that the inactivation of the proliferation (ERK) and pro-survival (AKT) pathways along with caspase-3 activation, PARP cleavage, and consequently DNA damage account, at least partly, for the anti-cancer effect of Frondoside A. Moreover, combination therapy either with oxaliplatin or 5-FU enhanced these effects and further clarified the capacity of Frondoside A to enhance the apoptosis induced mainly by 5-FU. The data also suggests that the inhibition of ERK1/2 phosphorylation with single treatments and its enhanced inhibition under the combination conditions suggests a potential impact of the treatments on colon cancer cell proliferation.

The dual inhibitory effect of our treatments on the PI3K–AKT and the Raf–MEK–ERK pathways is in line with the previously reported interactions between these two pathways [17]. Here, we demonstrate that the inhibition of AKT and ERK phosphorylation is crucial for the anti-tumor activity of Frondoside A. These results are in agreement with previous reports showing that Frondoside A inhibited AKT and ERK1/2 activation in TPA-stimulated breast cancer cells [18] and ERK1/2 activation in PGE2-stimulated breast cancer cells [19].

The current findings are in line with a study by Janakiran et al. demonstrating that Frondanol A5, a *Cucumaria frondosa* extract that contains several agents, including monosulphated triterpenoid glycoside Frondoside A, the disulphated glycoside Frondoside B, the trisulphated glycoside Frondoside C, 12-methyltetradecanoic acid, eicosapentaenoic acid, fucosylated chondroitin sulfate, and canthaxanthin/astaxanthin, inhibits HCT-116 colon cancer cell growth by inducing a G2 arrest followed by the induction of apoptosis [20].

This study provides sufficient rationale to carry out in vivo studies to confirm the relevance of the combination therapy. We believe that Frondoside A in combination with the standard cytotoxic drugs oxaliplatin and 5-FU currently used in colon cancer treatment may improve colon cancer therapy.

## Figures and Tables

**Table 1:** IC_50_ (µM) of Frondoside A compared to oxaliplatin and 5-fluorouracil at 48 h of treatment.

IC_50_ (µM)			
	Frondoside A	Oxaliplatin	5-Fluorouracil
HT-29	0.5	5	2.6
HCT-116	0.75	5	2.5
HCT8/S11	0.75	2.5	0.9
